# Comparison of the efficacy of endoscopic submucosal dissection and transanal endoscopic microsurgery in the treatment of rectal neuroendocrine tumors ≤ 2 cm

**DOI:** 10.3389/fendo.2022.1028275

**Published:** 2023-01-10

**Authors:** Rui Jin, Xiaoyin Bai, Tianming Xu, Xi Wu, Qipu Wang, Jingnan Li

**Affiliations:** ^1^ Department of Gastroenterology, Peking Union Medical College Hospital, Peking Union Medical College, Chinese Academy of Medical Sciences, Beijing, China; ^2^ Key Laboratory of Gut Microbiota Translational Medicine Research, Peking Union Medical College Hospital, Peking Union Medical College, Chinese Academy of Medical Sciences, Beijing, China

**Keywords:** rectal neuroendocrine tumor, endoscopic submucosal dissection, transanal endoscopic microsurgery, treatment, cohort study

## Abstract

**Introduction:**

Currently, complete tumor resection is considered the most effective treatment for rectal neuroendocrine tumors (NETs). Endoscopic submucosal dissection (ESD) and transanal endoscopic microsurgery (TEM) are recommended for rectalNETs ≤2 cm, but it is not clear which method is better. Thus, we evaluated the efficacy of ESD and TEM in the treatment of rectal neuroendocrine tumors (NETs) ≤ 2 cm.

**Methods:**

We conducted a single-centre retrospective cohort study between 2010 and 2021 of rectal NETs ≤ 2 cm in 114 patients with long-term follow-up data who were divided into ESD (n=55) and TEM groups (n=59). Our study assessed differences between groups in the complete resection rate of lesions, recurrence rate, surgical complications, procedure time, and length of hospital stay.

**Results:**

The co-primary outcomes were the complete resection rate of lesions and the recurrence rate. Compared to that in the ESD group, the complete resection rate was significantly higher in the TEM group (91.5% vs. 70.9%, *p*=0.005). The median follow-up time was 22 months in our study, and the follow-up outcomes suggested that the rates of recurrence were 1.8% (1/55) and 6.8% (4/59) in the ESD and TEM groups, respectively, with no significant difference between the two groups. The secondary outcomes of the evaluation were surgical complications, procedural time, and length of hospital stay. The rate of complications (gastrointestinal bleeding and perforation) was low in both the ESD (7.3%, 4/55) and TEM (5.1%, 3/59) groups. No difference in hospitalization duration was observed between the two groups in our study. However, the procedure time was significantly shorter in the ESD group than in the TEM group (27.5 min vs. 56 min, *p*<0.001).

**Conclusions:**

Although the rate of complete resection in the TEM group was higher than that in the ESD group, there was no difference in recurrence rates between the two modalities during long-term follow-up. Depending on the qualities of the available hospital resources in the area, one of the two approaches can be adopted.

## Introduction

Neuroendocrine tumors (NETs) are considered to originate from the cells of the diffuse endocrine system. The gastrointestinal (GI) tract is one of the most common sites of NETs, including the stomach, small intestine, appendix, colon, and rectum (1). The small intestine, rectum and colon are the sites with the highest incidence of GI NETs. With a significant increase in morbidities due to rectal NETs, rectal NETs (17.7%) have overtaken small intestinal NETs (17.3%) as the most prevalent GI NETs (2). More than half of patients are diagnosed incidentally, which is attributed to the widespread use of endoscopic screening for colon cancer (3). Rectal NETs are usually small, rarely have symptoms, and are mainly in the anterior or lateral wall of the rectum above the dentate line (4, 5). Most rectal NETs are localized at diagnosis, and distant metastasis is rare (2-8%) (6). The treatment of rectal NETs depends on tumour size. For rectal NET lesions <1 cm, the risk of metastasis is less than 3% (7). The European Neuroendocrine Tumor Society guidelines recommend local resection by an endoscopic or with the transanal technique (7). Tumours between 1-2 cm in size without muscularis or lymphatic invasion can be removed by local resection (7). For rectal NETs ≥2 cm or between 1-2 cm with muscularis or lymphatic invasion or positive margins after local resection, radical surgery is recommended (8). However, a study found no difference in the rate of recurrence between patients with rectal NETs ≤2 cm with or without lymphatic invasion treated by local resection and those treated with radical surgery (9). It is generally accepted that, rectal NETs ≤2 cm with or without lymphatic invasion can be removed by local resection. The available options for local resection include endoscopic polypectomy, endoscopic mucosal resection (EMR), modified EMR, endoscopic submucosal dissection (ESD) and transanal endoscopic microsurgery (TEM) (10, 11). The advantage of ESD and TEM is that the rate of histological complete resection is higher than that of EMR, with a trend towards replacing EMR, especially in Asia (12). Compared to ESD, TEM can achieve full-thickness rectal resection and achieve a higher satisfactory complete resection rate (13). However, the TEM technique also has higher anaesthesia-related adverse events and postoperative morbidity (11). Most critically, TEM is not more effective over the long run than ESD (14). Currently, both ESD and TEM are commonly used techniques for the treatment of rectal NETs ≤2 cm, but there is no consensus on which of the two treatment options is better. Thus, we conducted a single-centre retrospective cohort study with long-term follow-up to compare the efficacy of ESD and TEM in the treatment of rectal NETs ≤2 cm.

## Methods

### Study design

**Figure 1:**
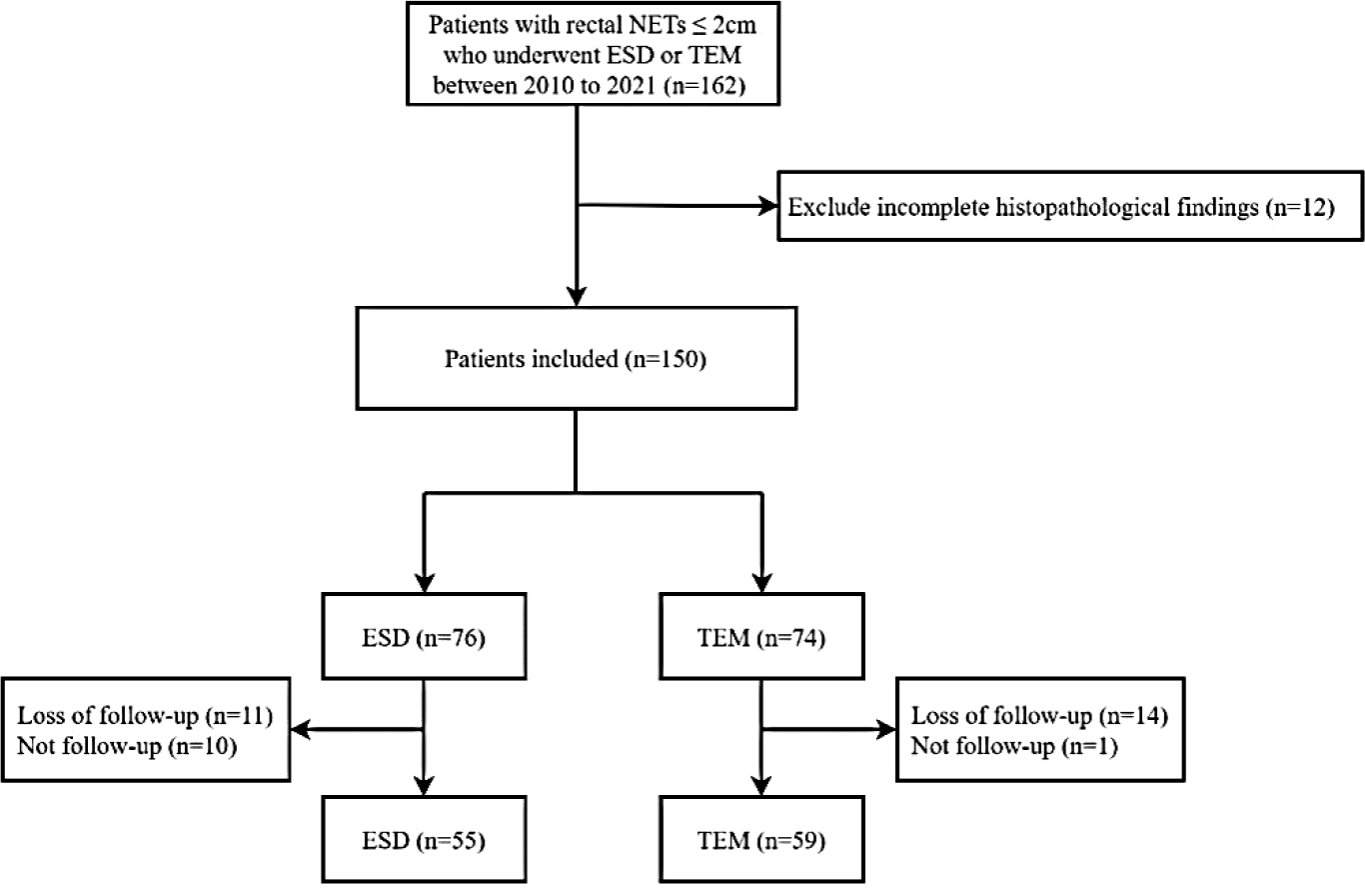
Flow chart for patient selection and follow-up.

This study consecutively included 162 patients with rectal NETs ≤2 cm treated with ESD or TEM at Peking Union Medical College Hospital, a tertiary hospital in Beijing, between June 2010 and June 2021. Clinical information, including the patients’ baseline data, tumour characteristics, pathological findings, and postoperative status, was collected from each patient through the electronic medical information system. Twelve patients with incomplete pathological findings were excluded. Then, 150 patients were divided into two groups and followed up. Thirty-six patients who were lost to follow-up or had no follow-up were excluded. Finally, a total of 114 patients were eligible for this study and were divided into ESD (55 patients) and TEM groups (59 patients). The study was approved by the Ethics Committee of Peking Union Medical College Hospital (No. K1331R). (The flowchart is shown in **Figure 1**).

### Treatment procedure

We routinely performed an ultrasound endoscopy or rectal ultrasound to assess tumour size, depth of invasion and pararectal lymph node metastases before ESD or TEM. For the 1-2 cm rectal NETs, computed tomography (CT) or magnetic resonance imaging (MRI) was performed before treatment as a clinical requirement.

All ESD procedures were performed by an experienced endoscopist team, who had completed over 5,000 cases for colonoscopy training and completed more than 300 cases for ESD training. After marking the border with a Dual knife, the submucosa was adequately injected with an injection solution. The mucosa was incised along the anal side, and the lesion was lifted along the submucosa until complete excision was achieved, with electrocoagulation of the wound to stop the bleeding without causing significant muscle damage.

TEM was also required to be completed by an experienced surgeon who had completed over 300 cases for TEM training. After the successful administration of general anaesthesia, the patient was placed in the prone position, the skin in the routine surgical area was sterilized, and sterile towels were laid. After dilation of the anus to approximately two fingers wide, a proctoscope was slowly inserted, the submucosal nodules of the rectum were found, and the proctoscope was fixed on the surgical bed. The back panel of the proctoscope was covered, the mirror tube was inserted, various tubes were connected, CO_2_ gas was introduced into the rectum, and the air pressure was regulated to between 12-15 mmHg. The rectum was observed for submucosal nodules with a smooth, yellowish-white surface mucosa. The submucosal nodules were gradually removed along the marker line from right to left, from superficial to deep, along with the entire intestinal wall. Then the rectal wound was sutured and checked for hemorrhage from the wound.

### Histopathological assessment

The data of all resected lesions were recorded by one pathologist specializing in gastrointestinal tumours, including tumour size, the status of the cut margins, depth of invasion, lymphovascular invasion, Ki-67 index and mitotic count. The pathological reports were reviewed by another experienced pathologist.

### Resection criteria

Lesions with negative lateral and deep margins were considered completely resected (The negative margin defined as no tumor cells contained). Conversely, incomplete resections were defined as lesions with positive lateral or deep margins.

### Definition

The procedure time of the ESD was defined to be from the insertion of the endoscope to the removal of the submucosal nodules. The operation time for the TEM was defined to be from the insertion of the rectoscope to the end of the sutured.

The rectal NETs were graded according to 2010 World Health Organization (WHO) classification diagnostic criteria: G1: Ki-67 ≤ 2% and/or mitotic count <2 per 10 high-power fields (HPF); G2: Ki-67 3-20% and/or mitotic count 2-20 per 10 HPF; G3: Ki-67>20% and/or mitotic count >20 per 10 HPF.

Local recurrence was defined as the development of NETs adjacent to previous scars at least 3 months after resection. Distant recurrence was defined as the development of NETs outside the rectal wall. Overall recurrence included local recurrence and distant recurrence.

### Follow-up

Outpatient examinations, telephone and email follow-ups were performed. The last assessment colonoscopy combined with CT/MRI was used as the cut-off time for follow-up. Those who did not complete the above examinations and could not be contacted by the researchers were considered lost to follow-up.

### Statistical analysis

SPSS 26.0 statistical software (International Business Machines Corporation Inc, New York, USA) was applied to analyse the data. Normally distributed continuous variables are expressed as the mean ± standard deviation, and a two-sample *t* test was used to compare the differences between the two groups. Non-normally distributed continuous variables are expressed as medians, and the Mann‒Whitney U test was used to compare the outcomes between the two groups. Categorical variables are expressed as frequencies and percentages, and the χ2 test or Fisher’s exact test was used for comparisons of ESD and TEM. *p*<0.05 was considered statistically significant. Recurrence-free survival for ESD and TEM was *calculated* using the Kaplan‒Meier curve, and the analysis software was GraphPad Prism 9 (GraphPad Software Inc, California, USA). Univariate and multivariate analyses were performed using the Cox proportional hazard model, and variables with P<0.05 in the univariate analysis were included in the multivariate analysis.

## Results

### Patient baseline data and tumour characteristics

In this study, 55 and 59 patients were eventually included in the ESD and TEM groups as determined through doctor‒patient communication, respectively. Baseline features (age, sex, history of smoking, alcohol consumption, diabetes mellitus, hyperlipidaemia, personal and family malignancy history, and previous EMR history), as well as tumour characteristics (number, location, size [diameter], depth of infiltration, and lymph node infiltration), are shown in **Table 1**. There was no difference between ESD and TEM in baseline features and tumor characteristics.

**Table 1:** Patients baseline data and characteristics of tumors.

Variable	ESD	TEM	*P* value
N	*55*	59	
Age at diagnosis(y, mean ± SD)	*52.9 ± 11.7*	51.1 ± 12.1	0.429
Sex (F/M)	*35/20*	41/18	0.508
History of smoking (%)	*23 (41.8)*	18 (30.5)	0.209
History of alcohol consumption (%)	*21 (38.2)*	14 (23.7)	0.095
Diabetes mellitus (%)	*5 (9.1)*	7 (11.9)	0.630
Hyperlipidaemia (%)	*4 (7.3)*	7 (11.9)	0.407
Combined malignancy (%)	*6 (10.9)*	8 (13.6)	0.667
History of malignancy in family members (%)	*14 (25.5)*	9 (15.3)	0.175
Previous EMR history	*2 (3.6)*	5 (8.5)	0.493
Number of tumors			0.768
Single lesion	*52 (94.5)*	55 (93.2)	
Multiple lesions	*3 (5.5)*	4 (6.8)	
Distance of the tumor from the anal verge (cm, median, range)	*8 (3-15)*	7 (3-10)	0.106
Tumour size			
Endoscopic evaluation (mm, median, range)	*6 (3-20)*	6 (2-20)	0.476
Histopathological evaluation (mm, median, range)	*7 (2-20)*	6 (2-20)	0.431
Depth of invasion			0.388
Mucosa (%)	*9 (16.4)*	6 (10.2)	
Submucosa (%)	*45 (81.8)*	50 (84.7)	
Muscularis propria (%)	*1 (1.8)*	3 (5.1)	
Plasma (%)	*0 (0.0)*	0 (0.0)	
Lymph node invasion (%)	*1/55 (1.8)*	1/56 (1.7)	0.99

SD, standard deviation; F/M, Female/Male; EMR, endoscopic mucosal resection.

### Treatment outcomes of ESD and TEM

Regarding efficacy, the complete resection rate was significantly higher in the TEM group than in the ESD group (91.5% vs. 70.9%, *p*=0.005). There were 16 cases of incomplete resection in the ESD group and 5 cases in the TEM group. The four patients with incomplete resection without lymphovascular invasion in the ESD group were treated with TEM. The two incomplete resection patients with lymphovascular invasion received low anterior resection (LAR) as salvage treatment. Two of five patients with incomplete resection in the TEM group received LAR.

Regarding safety, GI bleeding occurred in three patients in each of the two groups, and GI perforation occurred in one patient in the ESD group. There was no difference in complications between ESD and TEM (7.3% vs. 5.1%, *p* = 0.924). No difference was seen between the two groups in the days of hospitalization. The procedure time of ESD (27.5 min, range 10-60 min) was significantly shorter than that of TEM (56 min, range 20-180 min) (*p*<0.001) (**Table 2**).

**Table 2:** Treatment outcomes for ESD and TEM group.

Variable	ESD	TEM	*P* value
N	*55*	59	
Complete resection (%)	*39 (70.9)*	54 (91.5)	0.005
Additional salvage treatment	*6 (10.9)*	2 (3.4)	0.229
TEM (%)	*5 (9.1)*	0 (0.0)	0.056
LAR (%)	*1 (1.8)*	2 (3.4)	1.000
Complication (%)	*4 (7.3)*	3 (5.1)	0.924
Bleeding	*3 (5.5)*	3 (5.1)	1.000
Perforation	*1 (1.8)*	0 (0.0)	0.226
Hospitalization (days, median, range)	*4 (2-26)*	4 (1-9)	0.695
Procedure time (min, median, range)	*n=14, 27.5 (10-60)*	n=49, 57 (20-180)	<0.001

TEM, transanal endoscopic microsurgery; LAR, low anterior resection.

### Postoperative pathological assessment and tumor grade

There were 3 cases of lymphovascular invasion in the ESD group and 1 case of lymphovascular invasion in the TEM group. Regarding the Ki-67 index assessment, a Ki-67 index ≤2 was observed in 52 cases in the ESD group and 51 cases in the TEM group, and a Ki-67 index of 3-20 occurred in 3 cases in the ESD group and 7 cases in the TEM group. No cases in the ESD group had a Ki-67 index >20% from pathology, and 1 case in the TEM group had a Ki-67 index >20%. No differences were seen between ESD and TEM in the postoperative pathological assessment, including lymphovascular invasion and Ki-67 index. The grade of rectal NETs was not significantly different between the ESD and TEM groups (*p*=0.284) (**Table 3**).

**Table 3:** Post-operative pathological assessment and tumor grade.

Variable	ESD	TEM	*P* value
N	*55*	59	
Lymphovascular invasion (%)	*3 (5.5)*	1 (17)	0.561
Ki-67 (%)			0.324
≤2	*52 (94.5)*	51 (86.4)	
3-20	*3 (5.5)*	7 (11.9)	
>20	*0 (0.0)*	1 (1.7)	
Grade of WHO			0.284
G 1	*47 (85.5)*	45 (76.3)	
G 2	*8 (14.5)*	13 (22.0)	
G 3	*0 (0.0)*	1 (1.7)	

WHO, World Health Organization.

### Follow-up outcomes of ESD and TEM

The median follow-up time was 22 months (range: 2-117). In the ESD group, the median follow-up time was 19 months (range: 2-75). The median follow-up time was 28 months (range: 2-117) in the TEM group. One patient in the ESD group had local recurrence. No local recurrence was seen in the TEM group. Distant metastases occurred in 4 cases in the TEM group. The rates of overall recurrence were 1.8% and 6.8% in the ESD and TEM groups, respectively, with no significant difference between the two groups (**Table 4**).

**Table 4:** Follow up for ESD and TEM group.

Variable	ESD	TEM	*P* value
N	*55*	59	
Follow-up time (months)	*19 (2-75)*	28 (2-117)	0.012
Recurrence			0.119
No (%)	*54 (98.2)*	55 (93.2)	
Local recurrence (%)	*1 (1.8)*	0 (0/0)	
Distant metastases (%)	*0 (0.0)*	4 (6.8)	

All recurrences were observed in patients with complete lesion excision. There was no recurrences in patients with lesions considered incompletely resected, regardless of whether additional surgical treatment was provided, in the both of two groups.

**Figure 2:**
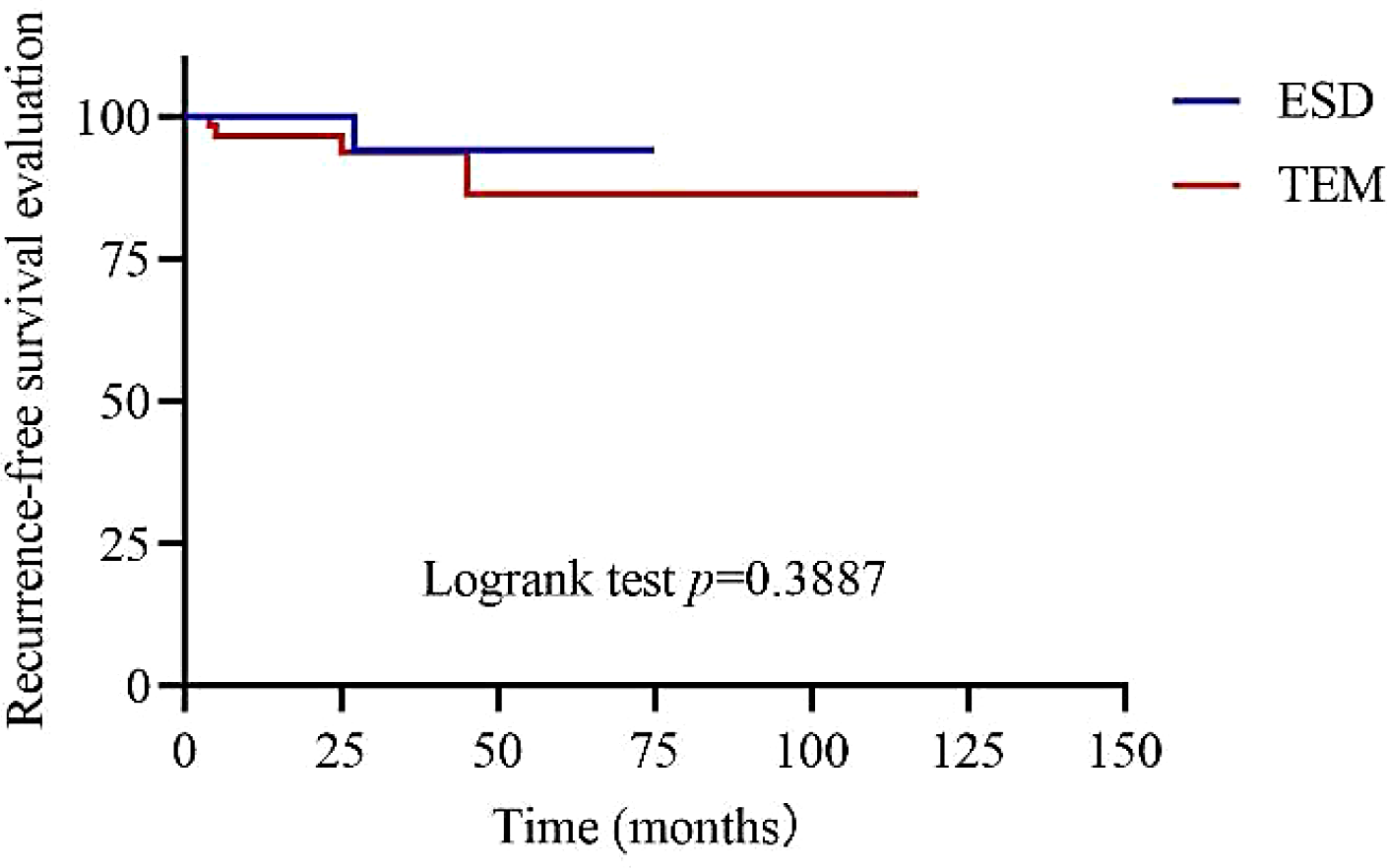
Recurrence-free survival time of patients with rectal NETs ≤ 2 cm between ESD and TEM groups.

**Figure 2**. showed the recurrence-free survival time of patients with rectal NETs ≤2 cm in both ESD and TEM groups. Univariate Cox analysis revealed that baseline hyperlipidaemia (hazard ratio [HR]: 11.152, 95% confidence interval (CI): 1.721-72.282, *p*=0.011), depth of invasion (HR: 8.280, 95% CI: 1.027-66.754, *p*=0.047), and distance of the tumor from the anal verge (HR: 0.327, 95% CI: 0.136-0.778, *p*=0.013) were associated with recurrence outcomes.

### Characteristics of tumors for patients with recurrent rectal NETs

The five patients with recurrent rectal neuroendocrine tumors were all male, and the median age was 44 years, ranging from 26-69 years. The median tumor diameter was 10 mm, ranging from 5-15 mm. Four patients had lesions invading the submucosa, and 1 patient had a lesion invading the muscularis propria. Lymph node invasion was observed in one patient. According to the WHO tumor grade, G1 tumors occurred in 4 cases, and a G2 tumor occurred in 1 case. One patient who underwent ESD was found to have recurrence *in situ* during follow-up. Four patients underwent TEM, and distant metastases were found at follow-up. The information on recurrence in these 5 patients with rectal NETs is summarized in **Table 5**.

**Table 5:** Characteristics of tumors for patients with recurrent rectal NETs.

Variable	1	2	3	4	5
Sex	M	M	M	M	M
Age	69	26	62	38	44
Tumor size (mm)	5	14	8	15	10
Depth of invasion	Submucosa	Submucosa	Submucosa	Muscularis propria	Submucosa
Lymph node invasion	No	No	No	No	Yes
Grade of WHO	1	2	1	1	1
Resection type	ESD	TEM	TEM	TEM	TEM
Margin invasion	No	No	No	No	No
Lymphovascular invasion	No	No	No	No	No
Type of recurrence	Local recurrence	Distant metastases	Distant metastases	Distant metastases	Distant metastases
Location of recurrence	Rectum	Liver	Liver	Lymph node	Lymph node
Time of recurrence (month)	27	45	25	5	4
Outcomes after treatment	ESD	Not available	Not available	LAR	Somatostatin analogues

ESD, endoscopic submucosal dissection; TEM, transanal endoscopic microsurgery; LAR, low anterior resection; WHO, World Health Organization.

## Discussion

In this research, we evaluated the effectiveness of TEM and ESD in the management of rectal NETs under 2 cm. The complete resection rate of lesions and the recurrence rate following treatment during long-term follow-up were the two metrics we used to assess efficacy. The full resection rate in the TEM group was much higher than that in the ESD group (91.5% vs. 70.9%). In our investigation, the median follow-up period was 22 months, and the follow-up results indicated that the recurrence rates in the TEM and ESD groups were 6.8% (4/59) and 1.8% (1/55) respectively. Four individuals in the TEM group and one patient in the ESD group among the patients with recurrent rectal NETs both suffered distant recurrence. Differences in surgical complications, procedure time, and length of hospital stay between the ESD and TEM groups were the evaluation’s secondary outcomes. Both the ESD (7.3%, 4/55) and TEM (5.1%, 3/59) groups had modest rates of problems. In our investigation, there was no difference in the length of hospitalization between the two groups. However, the ESD group’s method took far less time than the TEM group did (27.5 min vs. 56 min).

Traditionally, incomplete resection of the lesion is a factor for poor prognosis, and the goal of local excision is complete resection of the lesion. In our study, the complete resection rate in both the ESD and TEM groups was high, especially in the TEM group. Some studies have also confirmed this result. Sung et al. reported that both ESD and TEM achieved a high complete resection rate in T1 rectal NETs. The study further used propensity score matching and suggested that the rate of complete resection was higher in TEM than in ESD (92.3% vs. 71.2%) (15). Joon et al. found that the complete resection rate of rectal NETs was higher in the TEM group (14/14, 100%) than in the ESD group (19/23, 82.6%). No local recurrence of tumors was seen in any patient, regardless of complete or incomplete resection (16). Unfortunately, the sample size of the study was too small to confirm whether recurrence of rectal NETs was associated with complete resection of the lesion. In our study, no recurrence was seen in any patients with lesions considered incompletely resected. Therefore, we inferred that whether the resection margin of tumor cells was positive was not associated with tumor recurrence. Chung et al. detected thirteen (3.9%) patients with rectal NETs that presented positive resection margins after treatment with EMR, modified EMR and ESD. Five of thirteen patients accepted additional treatment, but no recurrence was observed in the patients with positive margins, with or without additional treatment (17). Similarly, Pattarajierapan et al. also found that 2.2% of rectal NET patients with positive margins had no recurrence (18). Li et al. reported that 54 patients had incompletely resected lesions out of 428 patients with rectal NETs, and the incomplete resection rate was 12.6%. All patients with rectal NETs underwent treatments including EMR, precutting EMR and ESD. No recurrence of the tumors was observed in the patients with incomplete resection during the follow-up period (19). On the whole, positive lesion margins do not indicate tumor recurrence. The necessity of additional treatment in patients with incomplete lesion excision is debatable. The above studies, including our study, suggest that endoscopic monitoring can be performed for rectal NET patients with incomplete lesion resection rather than additional treatment.

In terms of safety, there was no difference between the ESD and TEM groups in complications, including GI bleeding and perforation, or length of hospitalization. However, the procedure time was significantly shorter in the ESD group than in the TEM group. Compared to ESD, TEM operation needed additional suturing of the intestinal wall, which may extent the procedure time. Moreover, some studies such as Jung et al. and Mao et al. had defined the operation time different, which may cause bias in the procedure time (20, 21).

In previous studies, a number of factors, including tumor size, depth of invasion, lymphatic invasion, presence of central depression, positive resection margin, mitotic rate, and Ki-67 index, were found to predict unfavourable outcomes (22–25). In our study, univariate Cox analysis found that depth of invasion, the distance of the tumor from the anal verge and hyperlipidaemia were correlated with recurrence of the tumor. It has been shown that the depth of infiltration is associated with tumor recurrence, which is consistent with previous studies. Surprisingly, tumor distance from the anus verge and hyperlipidaemia were associated with tumor recurrence. Duan et al. reported that colorectal NET patients with lesions> 5 cm from the anal margin in the rectum have a better prognosis (26). This result may be associated with rectal vascularity and lymphatic distribution. There are few studies on the distance of the lesion from the anal verge affecting tumour recurrence, and this could be further investigated in the future. The relationship between hyperlipidaemia and the recurrence of rectal neuroendocrine tumours is unclear, but a study found that rectal NETs are more likely to occur and persist in areas with high serum cholesterol levels (27).

There was 1 patient and 4 patients in the ESD and TEM groups, respectively. Local recurrence, despite not significantly different, was only seen in the ESD group. All distant recurrence were seen in the TEM group. One of the patients who had distant metastases with a tumor size 15 mm in diameter and muscular involvement received TEM initially. Five months after TEM, lymph node metastasis was found in the rectal mesenteric region and further LAR with lymphadenectomy was performed. No recurrence was observed after 6 months. The choice of local resection or radical resection for rectal NETs between 10 mm to 20 mm remains controversial. The ENETS guidelines recommend local resection for rectal NETs<20 mm with a low mitotic rate and no muscular involvement (7). In addition, Shigeta et al. found that there was no difference in recurrence rate between local resection and radical resection in rectal NETs patients with tumor size>10mm and lymphovascular invasion (9). Therefore, more evidence is needed to clarify whether local or radical resection is more appropriate for rectal NETs between 10-20mm.

There are two limitations in the study. First, there was only one centre included in the study, so the results were limited. Second, the follow-up time was not long enough. The median follow-up times in the ESD and TEM groups were 19 and 28 months, respectively. Patients with incompletely resected lesions were followed up for 28 months. Patients with completely resected lesions were followed up for 27 months. The follow-up period was not long enough to strongly indicate that there would not be any recurrences in the future. In future studies, the follow-up time can be extended to further confirm that the recurrence rates after ESD and TEM are similar.

## Data Availability

The original contributions presented in the study are included in the article/supplementary material. Further inquiries can be directed to the corresponding author.

## Conclusion

Despite the fact that the TEM group had a greater percentage of full resection than the ESD group did, there was no difference in the rates of tumor recurrence between the two modalities during long-term follow-up. One of the two ways can be employed depending on the characteristics of the local hospital resources that are accessible.
